# Changing attitudes towards obesity – results from a survey experiment

**DOI:** 10.1186/s12889-017-4275-y

**Published:** 2017-05-02

**Authors:** C. Luck-Sikorski, S. G. Riedel-Heller, J. C. Phelan

**Affiliations:** 1 Institute of Social Medicine, Occupational Health and Public Health (ISAP), University of Leipzig, Philipp-Rosenthal-Str. 55, 04103 Leipzig, Germany; 2 Leipzig University Medical Center, IFB AdiposityDiseases, Philipp-Rosenthal-Str. 27, 04103 Leipzig, Germany; 3 SRH University of Applied Health Sciences, Neue Str. 28–30, 07548 Gera, Germany; 4 Mailman School of Public Health, Columbia University, 722 W 168th St, New York, NY 10032 USA

**Keywords:** Obesity, Public health, Stigmatization, Attitudes, Vignette study, Population-based study

## Abstract

**Background:**

This experimental study in a population-based sample aimed to compare attitudes towards obesity following three different causal explanations for obesity (individual behavior, environmental factors, genetic factors).

**Methods:**

The data were derived from an online representative sample. A random subsample of *n* = 407 participants was included. Two independent variables were investigated: cause of obesity as described in the vignette and cause of obesity as perceived by the participant regardless of vignette. Quality features of the vignettes (accuracy and bias of the vignette) were introduced as moderators to regression models. Three stigma-related outcomes (negative attitudes, blame and social distance) served as dependent variables.

**Results:**

Inaccuracy and bias was ascribed to the social environmental and genetic vignettes more often than to the individual cause vignette. Overall, participants preferred individual causes (72.6%). While personal beliefs did not differ between the genetic and environmental cause conditions (Chi^2^ = 4.36, *p* = 0.113), both were different from the distribution seen in the individual cause vignette. Negative attitudes as well as blame were associated with the belief that individuals are responsible for obesity (b = 0.374, *p* = 0.003; 0.597, *p* < 0.001), but were not associated with vignette-manipulated causal explanation. The vignette presenting individual responsibility was associated with lower levels of social distance (b = −0.183, *p* = 0.043). After including perceived inaccuracy and bias as moderators, the individual responsibility vignette was associated with higher levels of blame (emphasis: b = 0.980, *p* = 0.010; bias: b = 0.778, *p* = 0.001) and the effect on social distance vanished.

**Conclusions:**

This study shows that media and public health campaigns may solidify beliefs that obesity is due to individual causes and consequently increase stigma when presenting individual behavior as a cause of obesity. Public health messages that emphasize the role of social environmental or genetic causes may be ineffective because of entrenched beliefs.

## Background

Although the rise in prevalence rates of obesity in developed countries seems to be slowing down, over 1.4 billion adults are affected by this chronic condition [1]. This is reflected in rising obesity-related health care costs that challenge health care systems and make it a major public health concern [2]. The condition is associated with a major impact on health outcomes, including increased mortality in patients with more extreme levels of obesity [3]. Affected individuals, however, suffer not only from these somatic co-morbidities – their well-being is also affected by psychosocial factors.

One factor that may be associated with impaired health outcomes is perceived stigmatization and discrimination [4]. The stigma process includes the labeling of differences, negative attitudes, including blame, and social distance and exclusion [5]. Evidence for negative attitudes towards individuals with obesity is substantial [6] and has been shown in the general public [7] as well as various other settings. These negative attitudes include labeling individuals with obesity as lazy, indulgent and weak-willed [6]. Likewise, discrimination has been documented [8] and also seems to be increasing according to self-report [9]. Based on other areas of stigma research, we might expect the experience of being stigmatized to function as a mediating variable in the pathogenesis of obesity [10].

Reasons for the stigmatization of individuals with obesity have been discussed. Crandall and colleagues conclude that just-world beliefs, conservatism and a belief in the protestant work ethic contribute to obesity stigma [11]. According to these views, individuals with obesity are perceived as diametrical to values of healthy individuals, who are seen as contributing to society. Research shows that obesity is generally perceived as a self-inflicted condition that lies under an individual’s control [12]. The perception of controllability in turn was associated with higher levels of negative attitudes in several studies [13, 14]. This touches on a key focus of public health messaging about obesity. While prevention as well as intervention strategies often focus on individual behavior (eat less, exercise more [15]), this kind of messaging may contribute to the general public’s view of controllability [16]. The side-effects of such broad public health messaging on obesity stigma have not been investigated, but increasing calls for action have been made regarding a specific public health approach to combating obesity stigma [17]. This is of special importance as there is growing evidence that stigmatizing individuals with obesity does not motivate them to lose weight but rather decreases motivation and weight loss success [18].

Changing causal assumptions has been one starting point for anti-stigma efforts in smaller scale studies. A recent review of such studies reveals mixed results regarding their effectiveness in changing attitudes towards obesity. A variety of strategies to alter causal beliefs about obesity have been applied, including verbal and written presentations [19]. All studies in this review, however, are subject to limitations because of small and non-representative samples, ranging from 40 to 258 participants. One study reports results from a representative sample and evaluates the effect of giving genetic causal information on obesity [20]. The authors did not find an impact of providing genetic information on obesity stigma; however, a group receiving an individual based cause was lacking in this survey.

More alarming, there is emerging evidence that the narratives presented in public health messaging to induce policy support may affect not only policy support, but also may have effects on stigmatization [21]. In this particular study, participants read an article were the rising prevalence of obesity was framed as an “obesity epidemic” and public health crisis. This narrative increased stigmatizing attitudes in participants. Further research on the consequences of public health messaging is therefore needed.

The present experimental study in a population-based sample therefore aims to compare attitudes towards obesity following three different causal explanations for obesity (individual behavior, environmental factors, and genetic factors). It is hypothesized that participants who receive an individual based explanation for obesity will display the highest level of negative attitudes, while a genetic explanation will lower negative attitudes.

## Methods

The data were derived from a large online sample, recruited to be representative of the adult US population living in households with telephones. The study was part of the American National Election Studies (ANES) and invited participants aged 18 and above to complete online surveys. It has been described in detail elsewhere. The study was conducted during April and May 2009, and a total of 2409 participants completed the survey. The completion rate was 66%.

### Ethics

Knowledge Networks, the conducting institute of this study, adheres to the mandated Code of Standards and Ethics for Survey Research [22]. Additionally, the study was reviewed and approved by the Columbia University Medical Center Institutional Review Board. Participants signed up and gave consent to be contacted for the study by Knowledge Networks and were then informed that they had the right to withdraw consent at any time.

### Procedure

All respondents were randomly assigned to one of two different survey experiments. A total of 1850 participants were assigned to a vignette study presenting different causes for three conditions: coronary heart disease, depression and obesity. All participants were asked to read a written vignette (newspaper article) for one of these three conditions. After reading this newspaper like article, a set of questions regarding the article and the condition described in the article was presented. Participants received a small monetary incentive for participation.

### Sample

**Fig. 1:**
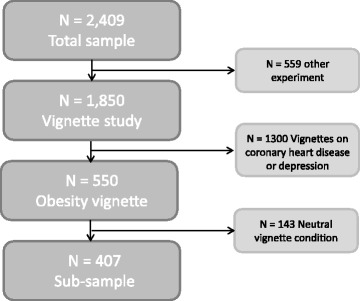
Sample flow chart

The present analyses only report on those participants who were assigned to the obesity condition (*n* = 550). Of these, *n* = 143 participants were randomly assigned to a neutral vignette, in which no causal attribution was given and participants were only informed that a new research project on obesity has started. Since no comparison to personal belief can be made in this sub-group and characteristics of the vignette cannot be included, we exclude these participants from our main analysis. However, we report all results in the full sample in Table 4. A random subsample of 407 participants completed the survey experiment with vignette manipulation on the cause of obesity (see Fig. 1 for a detailed sample flow chart).

Table 1 summarizes the sample characteristics and compares them to US 2010 census data for the adult population (aged 18 and older) [23]. It is evident that the sample is younger, under-samples certain ethnicities, and substantially differs in educational attainment from the general US population. Also, there was a substantial number of missing observations for self-reported BMI. In our subsample, *n* = 200 participants reported their height and weight (49%). Those who did indicate their BMI had higher BMIs on average compared to the US adult population. To take account of these differences, results are examined across age, education, ethnicity and gender groups by introducing interaction terms to the regression models. We also examined results across weight reported/weight missing groups and by reported weight for those who did report it. Additionally, we examined whether BMI moderated our results.

**Table 1:** Socio-demographics, sample *n* = 407

	Social environmental	Individual behavior	Genetics	*p* value^*^	Census Data^a^
Gender				0.170	
Male, n	63	62	47		49
(%)	45.7	45.3	35.6		
Age (n, Mean)	53.7	53.4	51.2	0.512	n/a
Age Categories				0.497	
18–44 (%)	26.8	24.8	33.6		48
45–64 (%)	49.3	47.5	45.0		35
65 and older (%)	23.9	27.7	21.4		17
Education				0.542	
No or below high school diploma, n	7	3	6		
(%)	5.1	2.2	4.9		14
Diploma and/or some college, n	58	65	53		
(%)	42.0	48.5	40.5		59
College Degree or PHD, n^b^	73	66	72		
(%)	52.9	49.3	55.0		27
Ethnicity				0.030	
White, Non-Hispanic	108	111	113		
(%)	78.3	81.0	86.3		72
Black, Non-Hispanic	15	16	3		
(%)	10.9	11.7	2.3		13
Other, Non-Hispanic	6	2	1		
(%)	4.4	1.5	0.8		12
Hispanic	8	6	10		
(%)	5.8	4.4	7.6		16
More than 2 races	1	2	4		
(%)	0.7	1.5	3.1		3
BMI^c^				0.313	
Normal-weight, BMI < 24.9	6	4	9		
(%)	8.6	5.6	15.3		31.6
Overweight, BMI 25–29.9	20	18	11		
(%)	28.6	25.4	18.6		33.9
Obese all classes, BMI > 30	44	49	39		
(%)	62.9	69.0	66.1		32.6
Obese Class I, BMI 30–34.9	16	22	17		
(%)	22.9	31.0	28.8		
Obese Class II, BMI 35–39.9	13	16	12		
(%)	18.6	22.5	20.3		
Obese Class III, BMI > 40	15	11	10		
(%)	21.4	15.5	17.0		

*p-values according to Chi Sq test for gender and education. Age differences were tested with a one-way ANOVA

^a^compared to US 2010 census data, for obesity census data from 2008 is reported

^b^includes Bachelor’s, Master’s and doctoral degrees

^c^BMI reported by *n* = 200 participants (49%)

### Independent variables

We examined two primary independent variables – cause of obesity as described in the vignette and cause of obesity as perceived by the participant regardless of vignette.

#### Experimentally manipulated cause

At the beginning of the survey, participants were asked to read a fictitious newspaper article. The vignette was presented in the form of a two-column article. Vignettes were derived from a previously conducted content analysis of newspaper articles and were similar in terms of length and prestige of sources cited [24]. Participants were randomly assigned to read one of three different explanations for obesity: social environmental (e.g. environmental causes), responsibility of the individual, and genetic causes. The social environmental vignette focuses on food availability, calorie laden food environments and time shortage as the primary causes of obesity.

Gender of the vignette subject was randomly assigned. After stating that the person described suffered from obesity, varying explanations were given, including expert statements by university professors (Department of Endocrinology, Columbia University). For example, in the vignette implying personal responsibility, the expert is quoted “People often make choices that directly lead to these problems even when healthy alternatives are possible”. In case of social environmental causes presented in the vignette, the expert states “Obesity results from taking in more calories than you expend, and the pace of life, combined with the calorie-laden food served in most restaurants, makes that harder and harder for many Americans to avoid”. Genetic causes are emphasized by providing this expert quote: “It’s essentially DNA roulette. Your parents give you a certain set of genes that can really raise your risk for conditions like this [obesity]”. The full text of the vignettes is available in Appendix. A manipulation check at the end of the questionnaire evaluated the correct recall of the cause presented in the vignette (Item: “To the best of your memory, the article you read placed the most emphasis on which of the following factors as the basic root cause of obesity?”).

#### Personal belief about cause

Participants were asked which factor (individual, environment or genetics) they personally believed was the most important cause for obesity.

### Potential confounding and moderating variables

We include age, gender and educational attainment as potential confounding variables. Educational attainment was classified in three categories: less than high school diploma, high school diploma and/or some college, and college degree or higher, including bachelor’s, master’s, and doctorate degrees. Additionally, variables covering the perceived accuracy of the vignettes were assessed and included in the analysis as moderating variables. Participants were asked to determine whether the vignette that was presented to them was biased and inaccurate (4-point Likert scale) and over- or underemphasized (5-point Likert scale) the presented cause. We conceptualize the accuracy and emphasis measures as indicating the degree of congruence between the explanation offered in the vignette and the participant’s pre-exisiting causal belief. As such, the accuracy and emphasis measures serve as a measure of the popularity of each explanation in the general population. In addition, they serve as potential moderators of the effects of the vignette messages. Specifically, a causal explanation that is readily accepted by a participant should have a different impact on his or her attitudes than an explanation about which the participant is skeptical. Thus we introduce interaction terms to the models for the accuracy and emphasis measures. We also use the variables in separate models as covariates.

### Dependent variables

Three different dependent variables were constructed to cover major areas of stigmatizing attitudes [5]. The negative attitudes scale was based on a semantic differential, including 11 adjective pairs (e.g. weak vs. strong, good vs. bad, likable vs. unlikable) and participants were asked to describe people with obesity on these dimensions (7-point scale). A mean score of negative attitudes was calculated. The Cronbach’s alpha reliability was α = 0.88. The scale was derived from previous psychiatric stigma research and constructed to fit all three conditions that were tested in the study (e.g. coronary heart disease and depression).

Furthermore, a blame scale was constructed, using three items on perceived responsibility and blame. These include having to blame no one but themselves, having done something wrong and being at personal fault for the condition. Again, a mean score of these items was determined. The scale shows a Cronbach’s alpha reliability of α = 0.76 for this sample. The final dependent variable covers the social distance dimension of stigma. This scale included two items that asked about not wanting to spend time with a person with obesity as well as not wanting to work with someone with the condition. Cronbach’s alpha of this scale was α = 0.79.

### Data analysis

Data was analyzed using STATA 13.1 [25]. The data were provided by the senior author of this article. They are openly available as part of the American National Election Survey (ANES). Chi Squared tests were used to assess differences between frequencies in categorical variables, while t-tests were used to detect differences in means. Linear regression models were conducted and the described dummy-coded independent variables were introduced to the model. Additional analyses were done to compare the causal explanation vignettes with the neutral vignette. Genetic causes were used as the reference category. Moderation was tested by introducing interaction terms (vignette by accuracy measure) to the models.

## Results

### Characteristics of vignettes

Table 2 summarizes responses to the vignettes across all three presented causes. Overall, the majority (86.2%) remembered the content of the vignette correctly. Recall differed by cause, however, with more participants remembering the genetic cause correctly. Of those who received the social environmental vignette, 17.2% remembered individual causes presented in the vignette. The same is true for those participants who received the genetic cause vignette – those who did not recall correctly, tended to believe that they had read an article on individual causation.

**Table 2:** Characteristics of Vignette

	Social environmental	Individual behavior	Genetics	Neutral Vignette	*p* value*
Vignette remembered correctly					0.001
Yes (%)	78.4	88.2	92.2	n/a	
Vignette biased and inaccurate					0.050
Strongly agree, (%)	10.3	3.7	6.9	7.1	
Agree (%)	25.7	23.0	37.7	27.0	
Disagree (%)	30.9	35.6	33.9	36.2	
Strongly disagree (%)	33.1	37.8	21.5	29.8	
Mean, SD	2.13, 0.99	1.93, 0.87	2.30, 0.89	2.11, 0.92	0.012^a^
Vignette content					0.001
Strongly overemphasized (%)	12.6	5.2	15.4	n/a	
Somewhat overemphasized (%)	23.7	11.2	35.9	n/a	
Right amount of emphasis (%)	56.3	69.4	45.4	n/a	
Somewhat underemphasized (%)	6.7	11.2	2.3	n/a	
Strongly underemphasized (%)	0.7	3.0	1.5	n/a	
Mean, SD	3.41, 0.82	3.04, 0.74	3.61, 0.83	n/a	<0.001^b^
Personal beliefs across vignettes					0.050
Social environmental (%)	16.9	10.4	12.3	13.4	
Individual Behavior (%)	70.6	80.7	66.2	70.4	
Genetics (%)	12.5	8.9	21.5	16.2	

*p-values according to Chi Square tests

^a^ANOVA, significant difference between individual cause and genetics vignette

^b^ANOVA, significant differences between social environmental and individual cause vignette as well as genetics and individual cause vignette

Although the majority of participants considered the vignettes non-biased (64.2%) and with the right amount of emphasis (57.0%), this means the acceptance of the vignette messages was far from perfect. Notably, perceived inaccuracy and bias varied by vignette. Inaccuracy and bias was ascribed to the social environmental and genetic vignettes more often than to the individual cause vignette. A similar picture can be seen for the question on whether the vignette placed the right amount of emphasis on the cause. Participants reported having received the right amount of emphasis across all three vignettes (57.1%), but higher rates in the individual cause vignette. Genetic cause was most often rated as being overemphasized.

Lastly, Table 2 presents concordance between the cause presented in the vignette and the respondents’ personal beliefs. Overall, participants preferred individual causes (72.0%), but most strongly in the individual behavior vignette (80.7%). While personal beliefs did not differ between the genetic and environmental cause conditions (Chi^2^ = 4.36, *p* = 0.113), genetic personal beliefs were different from the distribution seen in the individual cause vignette (Chi^2^ = 9.41, *p* < 0.001). The proportion of people in the individual behavior condition who state that they personally belief in self-inflicted causes of obesity is therefore significantly higher, compared to those that received the genetic explanation vignette. When the neutral vignette is included, we see that the proportion of people seeing obesity as a genetically caused condition is significantly higher in the genetic cause vignette compared to all others (Chi^2^ = 9.48, *p* = 0.023).

### Vignette manipulation and personal belief and associations with stigma variables

All regression models are presented in Table 3. Two outcomes, negative attitudes and blame, were not associated with any vignette manipulation but were associated with personal beliefs. Negative attitudes (b = 0.374, *p* = 0.003) as well as blame (b = 0.597, *p* < 0.001) increased with the belief that individuals are responsible for obesity. The cause presented in the vignettes, however, was not associated with these outcomes. By contrast, social distance was associated with the cause as presented in the vignette, but not with personal beliefs about cause. The vignette presenting individual responsibility was associated with lower levels of social distance (b = −0.183, *p* = 0.043). Across all three outcomes and both independent variables, female respondents reported lower stigmatizing attitudes (Table 3). Results remained unchanged when the neutral vignette was introduced to the models (Table 4). Across all participants (*n* = 550), the association between personal opinion and stigma-related outcomes remained as shown in Table 3, with one exception: The association of personal belief with individual causes (compared to genetic causes) was significantly associated to negative attitudes (b = 0.302, *p* = 0.028), potentially due to a larger sample to detect this difference.

**Table 3:** Linear Regression for the Outcomes Negative Attitudes, Blame and Social Distance, unstandardized Beta Coefficients and 95% Confidence Intervals

	Outcome negative attitudes				Outcome blame				Outcome social distance			
	Cause induced by vignette		Cause according to personal belief		Cause induced by vignette		Cause according; to personal belief		Cause induced by vignette		Cause according to personal belief	
	b	CI	b	CI	b	CI	b	CI	b	CI	b	CI
Age	−0.008^*^	−0.014; −0.002	−0.008^**^	−0.014; −0.002	−0.002	−0.006; 0.003	−0.002	−0.006; 0.003	0.002	−0.003; 0.007	0.001	−0.004; 0.006
Gender (ref Male)	−0.285^**^	−0.457; −0.113	−0.260^**^	−0.430; −0.090	−0.307^***^	−0.449; −0.165	−0.259^***^	−0.393; −0.125	−0.325^***^	−0.472; −0.178	−0.230^***^	−0.448; −0.152
Education												
No or below HS (ref.)												
HS/some college	0.030	−0.444; 0.503	0.063	−.0404; 0.530	−0.089	−0.461; 0.283	−0.046	−0.397; 0.304	0.136	−0.250; 0.522	0.125	−0.261; 0.511
College or higher	0.150	−0.321; 0.621	0.187	−0.279; 0.652	−0.082	−0.452; 0.288	−0.037	−0.386; 0.312	0.093	−0.291; 0.477	0.095	−0.290; 0.479
Cause												
Genetic (ref.)												
Social environmental	0.029	−0.178; 0.236	0.210	−0.109; 0.528	0.004	−0.166; 0.174	−0.097	−0.154; 0.347	−0.086	−0.262; 0.091	0.049	−0.227; 0.325
Individual behavior	0.109	−0.098; 0.317	0.374^**^	0.132; 0.617	0.163	−0.008; 0.334	0.597^***^	0.408; 0.787	−0.183^*^	−0.361; −0.006	0.163	−0.457; 0.373
Constant	4.527^***^		4.242^***^		2.766^***^		2.309^***^		1.681^***^		1.478^***^	
R^2^	0.052		0.074		0.057		0.163		0.058		0.054	
N	381		380		398		397		398		397	

^*^ *p* < 0.05, ^**^ *p* < 0.01, ^***^ *p* < 0.001

**Table 4:** Linear Regression for the Outcomes Negative Attitudes, Blame and Social Distance including neutral vignette, unstandardized Beta Coefficients and 95% Confidence Intervals

	Negative attitudes		Blame		Social distance	
	b	CI	b	CI	b	CI
Age	−0.007^*^	−0.011; −0.002	−0.002	−0.006; 0.002	0.002	−0.002; 0.006
Gender (ref. male)	−0.272^**^	−0.421; −0.123	−0.315^***^	−0.436; −0.194	−0.313^***^	−0.440; −0.189
Education						
No or below HS (ref.)						
HS/some college	−0.108	−0.513; 0.296	−0.177	−0.488; 0.134	0.074	−0.252; 0.400
College or higher	0.105	−0.297; 0.507	−0.186	−0.495; 0.123	0.075	−0.249; 0.400
Cause						
Neutral vignette (ref.)						
Social environmental	0.052	−0.152; 0.256	−0.118	−0.282; 0.046	−0.010	−0.182; 0.162
Individual behavior	0.138	−0.066; 0.343	0.043	−0.122; 0.207	−0.105	−0.278; 0.068
Genetic causes	0.023	−0.183; 0.229	−0.121	−0.287; 0.045	0.075	−0.100; 0.249
Constant	4.545^***^		2.891^***^		1.689^***^	
R^2^	0.059		0.060		0.052	
N	514		539		538	

^*^ *p* <0.05, ^**^ *p* <0.01, ^***^ *p* <0.001

### Vignette characteristics and associations with stigma variables

Adding the perceived accuracy of the vignette (bias and overemphasis) as covariates (Table 5) and moderators (Table 6) only slightly altered results. Both did not change the results for the outcome negative attitudes. We did not find any significant interaction for this outcome.

**Table 5:** Linear Regression for the Outcomes Negative Attitudes, Blame and Social Distance, including emphasis and accuracy as covariates, Unstandardized Beta Coefficients and 95% Confidence Intervals

	Negative attitudes		Blame		Social distance	
	b	CI	b	CI	b	CI
Age	−0.008^*^	−0.014; −0.002	−0.002	−0.006; 0.003	0.002	−0.003; 0.007
Gender (ref. male)	−0.261^**^	−0.436; −0.086	−0.303^***^	−0.448; −0.159	−0.296^***^	−0.445; −0.147
Education						
No or below HS (ref.)						
HS/some college	0.009	−0.465; 0.483	−0.082	−0.456; 0.290	0.119	−0.264; 0.502
College or higher	0.138	−0.333; 0.608	−0.079	−0.450; 0.292	0.092	−0.289; 0.473
Cause						
Genetic (ref.)						
Social environmental	0.017	−0.191; 0.225	0.020	−0.152; 0.192	−0.080	−0.257; 0.097
Individual behavior	0.109	−0.109; 0.326	0.199^*^	0.019; 0.379	−0.167	−0.352 to −0.018
Vignette characteristics						
Inaccuracy	0.050	−0.050; 0.150	0.023	−0.057; 0.105	0.123^**^	0.040; 0.207
Overemphasis	−0.059	−0.172; 0.054	0.034	−0.058; 0.126	−0.078	−0.172; 0.017
Constant	4.623^***^		2.592^***^		1.663^***^	
R^2^	0.055		0.063		0.078	
N	378		395		395	

^*^ *p* < 0.05, ^**^ *p* < 0.01, ^***^ *p* < 0.001

**Table 6:** Linear Regression for the Outcomes Negative Attitudes, Blame and Social Distance, introducing vignette characteristics, unstandardized Beta Coefficients and 95% Confidence Intervals

	Outcome blame			
	Inaccuracy		Emphasis	
	b	CI	b	CI
Age	−0.002	−0.007;-0.003	−.000	−0.006; 0.004
Gender (ref Male)	−0.281^***^	−.0424; −0.138	−0.296^***^	−0.439; −0.154
Education				
No or below HS (ref.)				
HS/some college	−0.095	−0.463; 0.272	−0.099	−0.468; 0.271
College or higher	−0.094	−0.460; 0.272	−0.114	−0.483; 0.254
Cause				
Genetic (ref.)				
Social environmental	0.015	−0.418; 0.448	−0.205	−0.943; 0.534
Individual behavior	0.778^**^	0.338; 1.328	0.980^**^	0.237; 1.732
Vignette characteristics				
Inaccuracy	0.120	−0.015; 0.256		
Overemphasis	--	--	0.092	−0.054; 0.238
Interaction Vignette characteristics				
^*^ Social environmental	0.006	−0.174; 0.185	0.676	−0.138; 0.273
^*^ Individual behavior	−0.294^**^	−0.490; −0.103	−0.250^*^	−0.469; −0.031
Constant	2.495		2.412	
R^2^	0.068		0.616	
N	397		395	

^*^ *p* < 0.05, ^**^ *p* < 0.01, ^***^ *p* < 0.001

Including the covariates and moderators did influence the effects of the vignettes on the other two outcomes. After including accuracy and bias as moderators, the individual responsibility vignette was associated with higher levels of blame (model including emphasis: b = 0.980, *p* = 0.010; model including bias: b = 0.778, *p* = 0.001, Table 6). For both moderators, the interaction terms were significant (emphasis*individual cause: b = −0.250, *p* = 0.026; bias*individual cause: b = −0.294, *p* = 0.003). The more the vignette was perceived as inaccurate and over-emphasizing; the lower was the level of blame for the individual responsibility vignette. The same pattern occurred with simultaneously including emphasis and bias as covariates: The vignette focusing on individual causes then was associated to higher blame (b = 0.199, *p* < 0.05).

After including the moderators, the vignette effect on social distance vanished (model including emphasis: b = −0.247, *p* = 0.531; model including bias: b = −0.157, *p* = 0.501); however, neither significant interaction effects (emphasis*individual cause: b = 0.006, *p* = 0.959; bias*individual cause: b = 0.001, *p* = 0.998), nor direct effects were found for this outcome, which is why we do not display data in Table 6. The perceived accuracy of the vignettes did not independently change the level of social distance sought by participants, but may have accounted for the small effect of the individual responsibility vignette we observed in the model without accuracy measures. The same was true when including accuracy and emphasis as covariates (Table 5). Overall, all other predictors remained significant as in the models in Table 3.

When introducing interaction terms to investigate the results across age, educational attainment, ethnicity and gender groups, we find no significant interaction terms for any dependent variables (data not shown). Additional analyses investigated whether results were affected by the gender of the person described in the vignette. The effects of the independent variables remained the same.

Conducting separate analyses in those participants with BMI reporting compared to those who did not report their height and weight, we find differences. The effects of individual behavior as a personal belief (b = 0.312, *p* = 0.119) on negative attitudes and the association of individual behavior as a cause in the vignette on social distance (b = −0.124, *p* = 0.355) vanished; however, this may be explained by issues of statistical power in this much smaller subsample. Furthermore, when including BMI as a continuous moderator, we find no significant interaction.

### Post-hoc analysis

Based on the reviewed literature, we decided to conduct a post-hoc analysis to test whether causal attributes to the individual was associated with higher stigmatizing attitudes compared to genetic and environment causal attribution. All regression models were re-run, using either the vignette presenting individual behavior as the cause of obesity or personal causal belief attributed to the individual as the reference category. Only attributing obesity to the environment based on personal beliefs was significantly associated with a lower level of blame (b = −0.500, *p* < 0.001). In all other regressions, there was no effect of causal attribution to the environment.

## Discussion

Negative attitudes towards people with obesity are highly prevalent in the general public [26]. Perceived stigmatization has a profound impact on the well-being of affected individuals and may contribute to impaired health outcomes in individuals with obesity [4]. Several attempts have been made to challenge these negative attitudes and make them a target of public health campaigning, but success in stigma reduction has been limited [19].

Based on findings that stigmatizing attitudes are rooted in causal explanation that point to responsibility of the individual, one approach addresses these causal explanations. This study therefore set out to investigate the effects of causal explanation, experimentally manipulated, on stigmatizing attitudes towards obesity. While we find only limited effects of the experimental vignette manipulation, personal beliefs of the participants were associated with higher levels of negative attitudes and blame regardless of the vignette read by the participant. After considering perceived bias and inaccuracy of the vignette as moderating variables, the individual behavior vignette was associated with higher levels of blame. Social distance was slightly lower in participants who received the individual responsibility vignette; however, after including perceived bias and inaccuracy of the vignette as moderators, this effect was no longer significant. Still, this finding points into an important direction that is known from research on mental health stigma.

Within attribution theory, a reduction of blame and hence an increased behavioral tendency to help and a lower desire for punishment are expected when a non-behavioral explanation for the condition is given. While social distance was not a behavioral outcome in the original attribution theory, it is considered a central part of the stigma process [5]. In mental health stigma research, a genetic attribution of causes of mental disorders led to higher desire for social distance (Phelan, 2005). This is where we see a fundamental difference of these two stigmata. We do not observe this pattern in our study, but on the contrary see a decline in social distance when a behavioral cause is presented. Social distance might be mitigated by behavioral explanations because people, unlike in regard to mental disorders, do not think there is danger of a genetic “contamination”. Another explanation may be that respondents feel that is it reversible and people can lose weight if a behavioral explanation is presented, leading to a lower desire for social distance. Social distance may then be influenced by other determinants than other components of the stigma process. Other central parts of the stigma process, negative attitudes and blame were investigated in this study as well. Contrary to our initial hypothesis, the vignette’s causal explanation for the most part did not affect attitudes towards the individuals with obesity.

By contrast, personal beliefs about cause were related to attitudes, and belief in individual causes was related to more negative attitudes. A recall check revealed that a large proportion of the participants remembered the content of the vignette correctly, hence eliminating quality issues of the vignette as a possible explanation for this finding. Considering other feedback variables regarding the vignette indicates a general preference for individual based causes. Compared to the vignette describing individual behavior causes, the other two vignettes were reported to have been more biased and inaccurate and regarded as putting too much emphasis on genetic and environmental causes. Together with the large number of participants that endorse individual causes for obesity (72.6%), this finding may reflect a general reluctance of the general public to embrace alternative explanations for obesity. The preference for individual based explanations for obesity has been shown consistently, even after vignette manipulation (e.g. [20]).

One reason for this persistent view may be media-based. Several studies show a prevailing level of stigmatizing attitudes displayed in public media [27, 28], accompanied by a consistent presentation of individual causation. Television formats such as “The biggest loser” may contribute to re-enforcing these beliefs. Yoo [29] proposes a theoretical framework explaining this association: Weight sensitized individuals who watch a show similar to this, experience a shift in locus of weight control and associate personal responsibility and more negative attitudes [29]. Taking into account that nowadays weight concern and a desire to remain normal-weight is common [27], media images affect large proportions of the population based on this model. In this study, concordant with previous research, the personal belief of individual responsibility of obesity was associated with higher levels of negative attitudes and blame towards the individual. This corresponds with recent findings. In a study where the effects of news reports on the “obesity epidemic” where investigated, the authors found that reporting on the effects that obesity has on the public health care system did, unintentionally, increase stigmatizing attitudes and weight bias [21].

In sum, it seems that the vignettes did not contribute to altering personal beliefs of the participants. This conclusion can be drawn since we would have expected some difference in personal beliefs according to vignette, but only found that in those who received the individual cause explanation, the personal belief of individual causes were more pronounced. This means that receiving an individual behavior vignette enhanced and confirmed the personal belief of individual causes of obesity, whereas any other vignette (genetics and social environmental) was not reflected in different personal beliefs across these two vignettes. This finding indicates that public health messages that focus on individual causes, or emphasize individual based intervention and prevention strategies, may even enhance beliefs of controllability and responsibility of individuals with obesity.

This is an important finding that adds to the emerging literature of careful public health messaging in obesity prevention and intervention. Simple messages that focus on the individual may harm more than they benefit and also contradict recent research results that emphasize the importance of environmental intervention strategies and facilitation of behavioral change [18]. Other findings come from a study where different causal explanations about obesity were compared within an experimental manipulation [30]. While biological explanations led to higher internalized stigma and personal responsibility explanations increased blame and weight bias overall, emphasizing the role of the (food) environment had no negative impact. Genetic explanations may seem easy to introduce to the public, but may come with negative side-effects. In particular, we observed a high number of participants that were willing to endorse genetic explanations after being presented with a vignette that emphasized genetics. Although not significant, potentially due to a lack of statistical power, this finding may warrant further research.

Introducing environmental causes on the other hand, may hence be an option for further public health campaigns, too. A very recent study supports this notion: Using extended narratives on blame on the individual vs. blame on the environment led to support of policy interventions that either penalized or protected people with obesity [31]. What defines an “extended narrative” however, still needs to be investigated; our study implies that these will need to be stronger in argument than the ones focusing on individual responsibility. One could argue that continued exposure to an explanation – as it had been the case with individual causes for obesity – can be a key to changing causal beliefs.

### Limitations

This study has some limitations. First, although it used a probability sample of the US adult population, due to differential response in different segments of the population, the sample does not perfectly represent the general population. We have addressed this limitation by running interaction analyses. We find that results did not differ across the demographic groupings, reducing our concern that a more representative sample would produce different results. Second, it would have been desirable to include personal BMI in the analyses. Previous studies show an association of BMI and stigmatizing attitudes [32] and causal beliefs may be different according to weight status. Due to a large number of missing values and the limitations of self-report measurement, we decided against including BMI. However, we decided to conduct post-hoc analyses to check whether inclusion of BMI in a smaller sample (*n* = 192) would alter our results. This was not the case; all results shown in Table 3 were reproduced. Furthermore, our sample size may not have been sufficient to detect all statistical differences (such as the differences in agreement to genetic causes shown in Table 2). For the main outcomes, however, we were able to detect statistically significant differences.

Although the vignettes were based on actual news articles, the vignettes’ lack of impact could be due to shortcomings in how engaging or compelling the vignettes were. We also find differences in perceived overemphasis and inaccuracy across the vignettes. This may be because of participants favoring the individual causes view, but may also in part reflect differences in the clarity and persuasiveness of the vignettes. Arguing against this explanation of the findings, however, are the facts that most people remembered them correctly and accepted their content as valid. Additionally, there has been one other study, showing the limited impact of vignette driven intervention [20].

Additionally, data were from a study carried out in 2009. Since then, a number of things have changed in the perception of obesity as a chronic disease (e.g. the American Medical Association’s decision to define obesity as a disease). However, several studies have demonstrated that negative attitudes towards obesity are still the same, and may be even rising [33]. Public portraits are still vastly negative [27] and interventions for obesity stigma have failed to show efficiency [19].

Lastly, we were not able to include measures of implicit weight bias. It has been shown that implicit weight bias exists [6], and that it – independently from explicit bias – may predict discrimination [34]. However, implicit and explicit measures only correlate moderately [35].

## Conclusions

In conclusion, this study shows that messages about obesity may contribute to weight stigma when presenting individual behavior as a cause of obesity. The same is true for public health messages that emphasize the role of individual behavior. Public health messages that emphasize the role of social environmental or genetic causes may not work because of entrenched beliefs. This point is underscored by our finding that, for two of the three outcome measures, the effect of the vignette was moderated by the degree to which participants believed the vignette was accurate and unbiased. To reduce stigma, we may need to focus on other instruments to alter causal attribution in obesity or even consider newer approaches, such as social consensus theory [36], which focuses on social consensus to express attitudes and to act based on certain beliefs about minority groups. By challenging the widespread notion that the stigmatization of individuals with obesity is something that is endorsed by the general public and generally accepted, attitudes are expected to change. Additionally, social environmental and genetic messages may not be as strong as they need to be to actually change attitudes – future studies ought to therefore focus on making them more persuasive.

## Appendix

### Headlines

Modern life condition: “Obesity linked to Pressures of Modern Life”.

Behavior condition: “Obesity linked to Individuals’ Own Choices and Behavior”.

Genetic condition: “Obesity linked to Genes”.

[Daniel/Katherine] Link is a 50-year-old postal worker in Des Moines, Iowa. Like many Americans, [Daniel/Katherine] is obese.

We all have a general idea of what obesity is – excess body weight in the form of fat. In recent years, obesity has been more precisely defined in terms of “body mass index,” or BMI, which is based on a person’s height and weight.

Obesity is defined as a BMI of 30 or more. That means someone who is 5-ft-4 is obese at 175 lb, while a person who is 6 ft tall is obese at 222 lb.

Obesity can contribute to health problems such as cardiovascular diseases and type 2 diabetes.

Experts point to our modern way of life as the main cause of conditions like those of [DANIEL/KATHRYN] Link.

Modern life in America is hectic and stressful. There are increasing pressures to perform and achieve at work and school, fueled by Americans’ desire to get ahead, by economic uncertainty, and in many cases, by the simple need to bring home enough money to put food on the table. In a typical family, both parents often end up working long hours, kids carry home backpacks full of homework, and there are just not enough hours in a day.

“Everyone is living in the fast lane, whether they like it or not,” said Dr. Bruce Firman, Professor of Endocrinology at Columbia University. “Most people would not choose this lifestyle; it is just what the modern world demands.” We are simply too busy – and too stressed. This, according to experts, is hurting the health of Americans in a variety of ways.

One consequence is obesity.

### Modern life

Many families don’t have time to fix a nutritious breakfast or dinner and are forced to stop at a restaurant or get take-out. “By the time we both get off from work, there simply is not time to prepare a home-cooked meal,” said [MR./MRS. LINK]. This is a big contributor to obesity, because restaurant food is high in calories and fat.

Fast food is the worst culprit, but it’s not just McDonald’s. Nearly every dish and beverage Americans now consume in restaurants is supersized. An average serving of pasta is now 480% greater than the one-cup recommended serving size.

The same time pressures mean that we have little time for exercise to burn off those calories. “Obesity results from taking in more calories than you expend, and the pace of life, combined with the calorie-laden food served in most restaurants, makes that harder and harder for many Americans to avoid,” said Dr. Firman.

### Behavior

Experts point to peoples’ own behaviors and habits as the main cause of problems like those of [DANIEL/KATHRYN] Link.

“People often make choices that directly lead to these problems even when healthy alternatives are possible,” said Dr. Bruce Firman, Professor of Endocrinology at Columbia University. “People lead sedentary lifestyles and choose their food poorly, leading many to exceed the calories they need,” said Dr. Firman.

Many people point to fast-food restaurants as the culprit in Americans’ overconsumption of calories. However, representatives of the restaurant industry point out that consumers want those calorie-laden products.

Wendy’s experienced the discrepancy between what people say and what they do last year when the chain put a fresh fruit bowl on the menu. Despite a $20 million marketing push, the fruit did not sell. “We listened to consumers who said they want to eat fresh fruit, but apparently when it comes down to it, they prefer the burgers,” said Denny Landon, a spokesman for Wendy’s.

Another problem is that many Americans, like [DANIEL/KATHRYN] Link, are “sedentary,” meaning they get less the 30 min of moderate exercise a day. “I know I should exercise, but it’s so hard to get motivated,” said [MR./MRS.] Link.

“It really comes down to the individual and the choices he or she makes,” said Dr. Firman. Until people take this message to heart, Americans will continue to suffer from conditions – like obesity – brought on by unhealthy lifestyles.

### Genetics

Experts point to genetic factors as the main cause of conditions like those of [DANIEL/KATHRYN] Link.

A person’s genetic makeup plays a powerful part in determining whether they become obese, said Dr. Bruce Firman, Professor of Endocrinology at Columbia University. “It’s essentially DNA roulette. Your parents give you a certain set of genes that can really raise your risk for conditions like this.”

It has long been known that genes play a role in causing obesity based on how the condition is distributed in families. Dr. Firman explained, “identical twins share 100 percent of their genes, while fraternal twins share only 50 percent of their genes (the same percentage as non-twin brothers and sisters). Therefore, to the extent that genes are influential, identical twins should be more alike than fraternal twins.” And that turns out to be true for obesity.

The genetic determinants of obesity work much differently than with eye color, which is caused by a single gene.

With something like obesity many genes work in concert with each other.

New advances in genetics are making it possible to identify some of the specific genes involved in obesity. One of these, known as INSIG2, regulates fatty acid and cholesterol synthesis. [DANIEL/KATHRYN] Link knows that [HE/SHE] has the form of the INSIG2 gene that leads to obesity.

“I’m not happy about that,” said [MR./MRS.] Link, “but it’s just something I’ll have to learn to deal with.”
